# Dataset of metals and metalloids in food crops and soils sampled across the mining region of Moquegua in Peru

**DOI:** 10.1038/s41597-023-02363-0

**Published:** 2023-07-25

**Authors:** Noelia S. Bedoya-Perales, Elias Escobedo-Pacheco, Diogo Maus, Alisson Neimaier, Guilherme Pumi

**Affiliations:** 1 Universidad Nacional de Moquegua; Calle Ancash s/n, Moquegua, 18001 Peru; 2 Instituto Federal Farroupilha; Alameda Santiago do Chile, 195 - Nossa Sra. das Dores, 97050-685 Santa Maria, RS Brazil; 3 Programa de Pós-Graduação em Estatística - Universidade Federal do Rio Grande do Sul, 9500 Bento Gonçalves avenue, 91509-900 Porto Alegre, RS Brazil

**Keywords:** Agriculture, Developing world, Sustainability, Environmental impact, Scientific community

## Abstract

In recent years, there has been an increase in interest in the accumulation of heavy metals and metal(loid)s (HMM) in areas where agriculture and mining exist side by side. As a contribution to this body of knowledge, we report the first dataset into HMM concentrations in food crops and agricultural soils in Moquegua, which is a typical mining region and contains one of Peru’s largest copper deposits. Thanks to its geographic diversity, samples were taken in different agroecological regions at altitudes between 9 and 3,934 m. For food crops, 31 elements were measured using inductively coupled plasma mass spectrometry and atomic absorption spectrometry. For soils, 23 elements were measured using inductively coupled plasma optical emission spectrometry. Thus, the dataset includes a total of 13,828 observations from 341 sampling sites. We hope that this dataset will facilitate a wide range of agricultural and food safety studies, as well as serving as a reference for monitoring changes in pollution over time or comparing HMM levels with other farmlands influenced by mining activities.

## Background & Summary

Heavy metal contamination of food crops and soils is a pervasive problem worldwide, arising from both human and natural activities that release these elements into the environment. Heavy metals can penetrate soil, enter groundwater, and accumulate in the food chain, where they can cause harm to the biota^1^. They can therefore become damaging to human health and ecosystems, depending on the concentration and exposure time^2,3^. However, heavy metals are not the only damaging elements, other metals and metalloids can be hazardous to plants by inducing symptoms of phytotoxicity^4–7^.

This problem is exacerbated in areas where mining and agriculture coexist, as studies have shown that soil and crop contamination is more severe than in districts without mining or located far from mining activities^8,9^. An excess of these elements in agricultural soils can reduce crop yields because of the risk of bioaccumulation and biomagnification in the food chain^10^, although the mechanism and intensity of absorption by plants varies by species, variety, altitude at which they are grown, soil characteristics and other factors^11,12^.

Comparing levels of heavy metals and metal(loid)s (HMM) with regulatory standards is one approach to assess the potential risk of these contaminants to human health and the environment. As far as food crops are concerned, the Codex Guideline Level is widely used as a reference, as well as being the basis for national regulations and the international trade in foodstuffs^13^. In the case of soils, national quality standards are commonly used as a preventive tool in environmental management. Even so, one important limitation is that these standards only include certain HMM and they differ from one country to another^14,15^. Nevertheless, when local studies of HMM levels are carried out, quantitative data is needed as a reference for the purpose of comparison. We can thus increase our understanding of the dynamics of concentration, distribution and sources of pollution, which also vary over time. Various studies have concluded, therefore, that HMM monitoring is an essential step in protecting the environment and human health^16,17^.

Data on the concentration of HMM in food crops in areas influenced by mining can be found in papers reporting the results of studies conducted in various countries in Europe^18,19^, Africa^8,20^, Asia^21^, and the Americas^22–25^. Other academic studies have also proved useful in that they provide ranges of HMM concentrations in food crops^4^ on a global scale, as well as databases for evaluating global soil health^26^ and global mining land use^27,28^. It is noteworthy, however, that there is a dearth of research and HMM data to assess the accumulation of HMM in farmlands on a regional scale.

The importance of carrying out further studies on the HMM concentrations in places close to copper deposits has already been highlighted in a study carried out in the Antofagasta region in the North of Chile (world’s largest copper producer)^29^. If future copper demand growth predictions are taken into account^30,31^, it will be necessary to document the evolution of HMM concentrations in the areas where copper deposits that are yet to be explored are found, such as southern Peru^28^. The challenge is even greater if one considers that, in general, the land areas of South America have been identified as one of the degradation hotspots in both mid- (2031–2060) and long-term (2071–2100) futures^32^. Given the challenges this poses for food and soil security, regional research about HMM pollution status becomes essential to develop action plans to manage environmental issues.

The objective of this investigation was to contribute to the existing body of work by providing primary data on HMM levels in food crops and soil samples collected in the department of Moquegua, located in south eastern Peru. Moquegua, the second smallest department in Peru, covering only 1.2% of the country’s area, offers an interesting study area due to its unique characteristics. Notably, it is home to one of the largest copper deposits in the country, contributing to Peru’s position as the world’s second-largest producer of the metal^33^. Agriculture in Moquegua is carried on from sea level to an altitude of at least 4,000 meters, in different agroecological regions producing different food crops^34^. That being so, these characteristics enable us to visualise the dynamics of HMM in relation to altitude.

As far as we know, this is the first dataset on HMM concentrations in food crops and agricultural soils for the department of Moquegua. The dataset provides results on HMM concentrations in samples collected at altitudes ranging from 9 to 3,934 meters above sea level (m.a.s.l), including a total of 13,828 observations and, for each sample, gives information on sample identification, geographic location of 341 sampling sites and the analysis method.

**Fig. 1:**
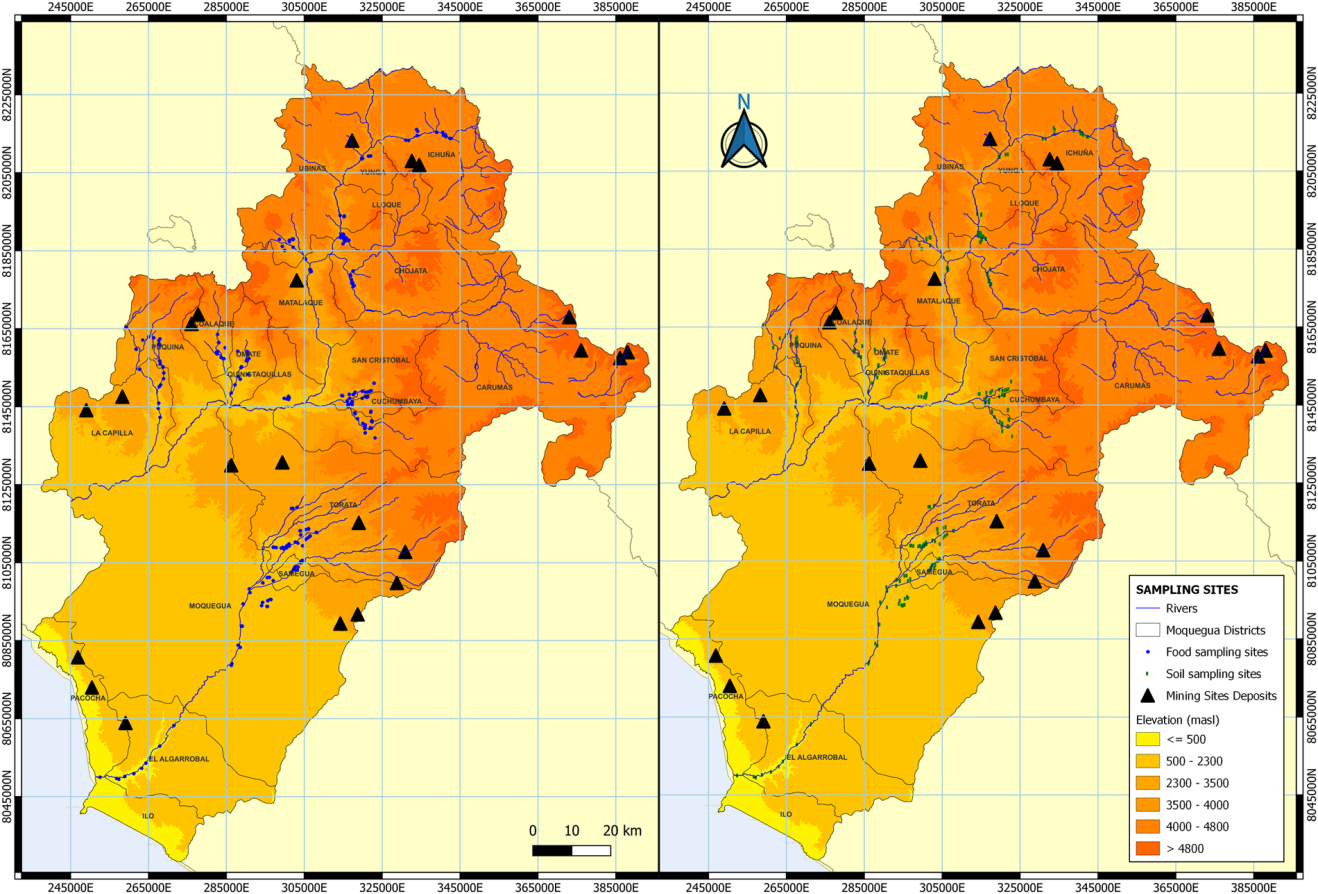
Agricultural areas with records of heavy metals and metal(loid)s (HMM) presented in the dataset. The figure shows the department of Moquegua and the location of the farming areas where samples of food crops (left side) and agricultural soils (right side) were collected. Altitudes varied from 9 to 3,934 m.a.s.l.

Figure 1 simplifies the scope of the investigation with recorded data on HMM in food crops and agricultural soils presented in the dataset.

The data collected in this study are useful for a wide range of audiences, including academics, public policy decision-makers, and individuals interested in the subject. These data can be used to compare HMM concentrations with samples from other agricultural areas affected by mining (especially from the copper exploration), using various variables such as sample type and altitude^24,35^. They can also be used for statistical analysis in order to evaluate sources of pollution^36^ or to characterise soils^21,37^. In another line of research, the dataset can be used to establish soil pollution indices^38^ or to determine ecological risk^39^. By determining the per capita consumption of the food crops in the local area, we can evaluate the health risk associated with exposure to toxic metals^40^ and establish regulatory reference values for public policy and food innocuity decisions^41,42^. Moreover, since the concentration of HMM can vary over time, this dataset serves as a valuable baseline for monitoring changes in pollution levels^43^ and assessing the effectiveness of remediation efforts work^44,45^ or changes in soil and crop management practices^14,46^. The complete dataset is available at Figshare 10.6084/m9.figshare.c.6572563.v1.

## Methods

### Geographic coverage

**Fig. 2:**
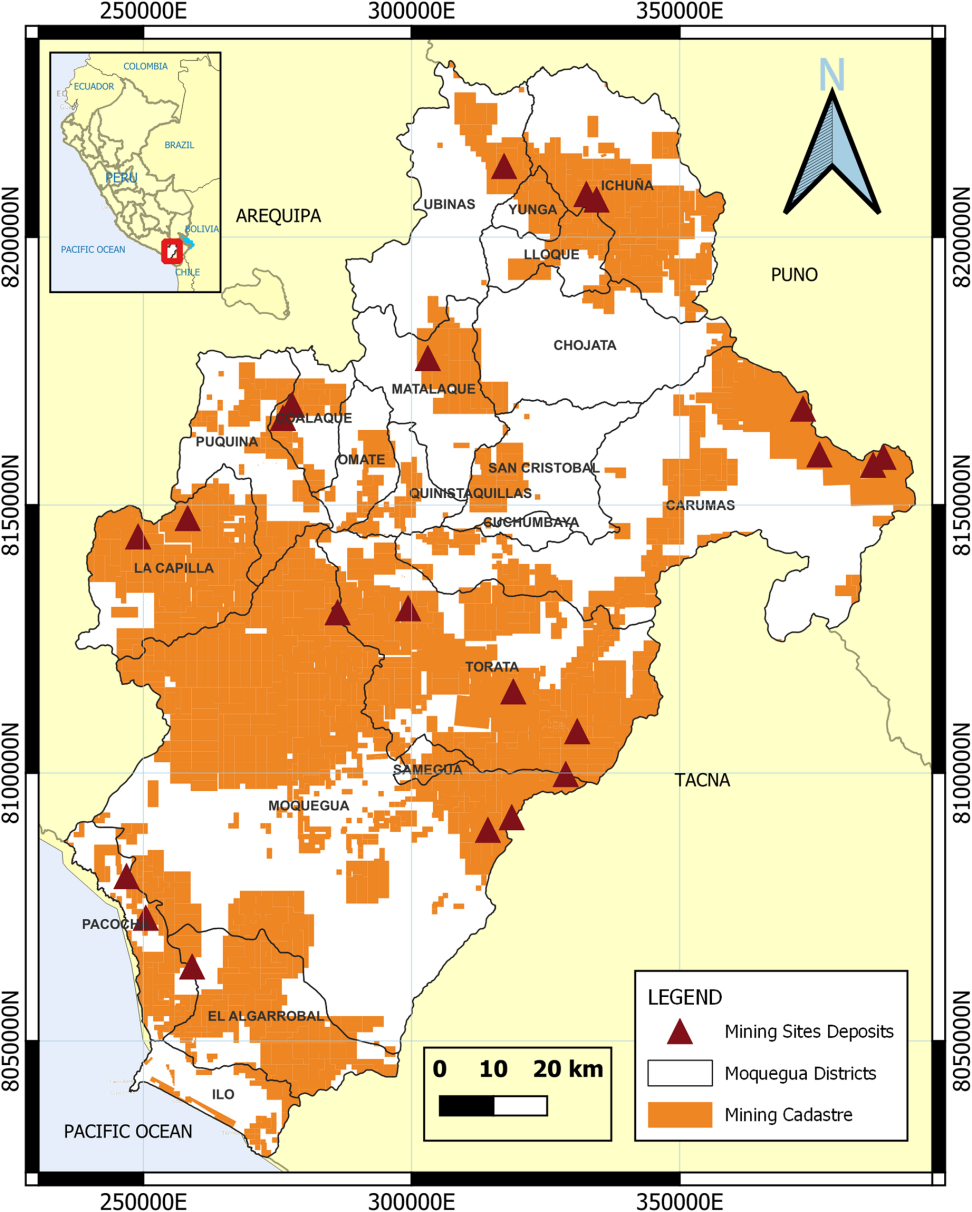
Location of the study area. Moquegua is the second smallest department in Peru, equivalent to 1.2% of the country’s territory (15,733.97 Km^2^), and large ore deposits have been discovered throughout the department. It consists of 20 districts grouped into 3 provinces: General Sánchez Cerro (Ichuña, Yunga, Lloque, Chojata, Ubinas, Matalaque, Coalaque, Omate, Quinistaquillas, Puquina, La Capilla), Mariscal Nieto (Moquegua, Samegua, Torata, Carumas, Cuchumbaya, San Cristóbal) and Ilo (Ilo, Pacocha and El Algarrobal).

The department of Moquegua is in southern Peru, between 15°17′ and 17°23′ latitude south; it covers 1.2% of Peruvian territory (15,733.97 km^2^)^47^. It has important ore bodies in much of its area (Fig. 2), which make a significant contribution to Peru’s position as the world’s second largest copper producer^33^.

The department of Moquegua contains a diversity of land use environments. It contains coastal and highland regions, at altitude varying from sea level to more than 6,000 m.a.s.l.^47^. This altitude range facilitates diversified agriculture at least up to 4,000 m.a.s.l.

Most agricultural units are characterized by covering less than 5 ha^48^, and small farmers face a range of water-related challenges. Peru generally suffers from water stress^49^ and in the department of Moquegua in particular, farming has resorted to ingenious forms of water management since ancient times, using canals, roads and terraces that are still employed today^50^. The limited availability of water for irrigation continues to be one of the greatest challenges for farmers, but there is an additional worry about pollution from agriculture and mining activities, which often becomes a driving factor for social conflicts^51,52^.

Given the lack of data on the HMM concentrations in soils and food, the study covers the three provinces into which the department is divided: General Sánchez Cerro, Mariscal Nieto and Ilo. These provinces represent 42.4%, 55.1% and 2.5%, respectively, of the land under cultivation^53^. As there has been no earlier monitoring of HMM levels in food crops or agricultural soils, participation by local farmers and technicians from the Regional Agriculture Office of Moquegua (DRA-Moquegua) was necessary to choose the 341 sampling sites due to convenience. To ensure a representative sample, we considered several factors, including altitude, harvest period, statements from farmers regarding the potential contamination of irrigation water by heavy metals, the significance of the crops to the local diet, and the availability of the most emblematic food crops in each province when fully ripe. Additionally, we obtained permission from landowners to collect samples from their farms.

In the province of General Sánchez Cerro, the sampling sites were situated between 1,539 and 3,934 m.a.s.l., and contained permanent and temporary crops. The first group included the avocado (*Persea americana*) and sweet lime (*Citrus limettioides*, locally known as *lima aromática de Omate)*. Andean tubers such as potato (*Solanum tuberosum* L.), mashua (or isaño) (*Tropaeolum tuberosum*), oca (*Oxalis tuberosa* Molina) and olluco (*Ullucus tuberosus Caldas*) were placed in the second group. Samples of faba bean (*Vicia faba*) and corn (*Zea mays L*. ssp amiláceo) were also collected. In two sectors of the districts of Matalaque and Chojata (Ánimas-Huarina and Pachas, respectively), farmers reported food shortages caused by contaminated irrigation water. For that reason, we included fields growing alfalfa (*Medicago sativa*).

The province of Mariscal Nieto is primarily known for its vegetable production. For this study, we selected sampling sites situated between 964 and 3,864 m.a.s.l., where only temporary crops were present: chard (*Beta vulgaris* var. Cicla), celery (*Apium graveolens*), spinach (*Spinacia oleracea*), beet (*Beta vulgaris*), white carrot (or yellow cassava), strawberry (*Fragaria vesca*), lettuce (*Lactuca sativa*), tomato (*Lycopersicon esculentum* Mill.), carrot (*Daucus carota*), corn (*Zea mays L*. ssp amiláceo), oca (*Oxalis tuberosa* Molina), mashua (or isaño) (*Tropaeolum tuberosum*) and potato (*Solanum tuberosum* L.). In the province of Ilo, some fruit crops are grown on small parcels totalling no more than 1 or 2 hectares. Some vegetables are also grown, but in such small quantities that they are not included in local farm statistics^53^. We therefore only collected potatoes as a temporary crop. Olives (*Olea europea*) were also collected as this is the only province in Moquegua where they are cultivated permanently. Thus, the sampling sites were situated between 9 and 357 m.a.s.l.

**Fig. 3:**
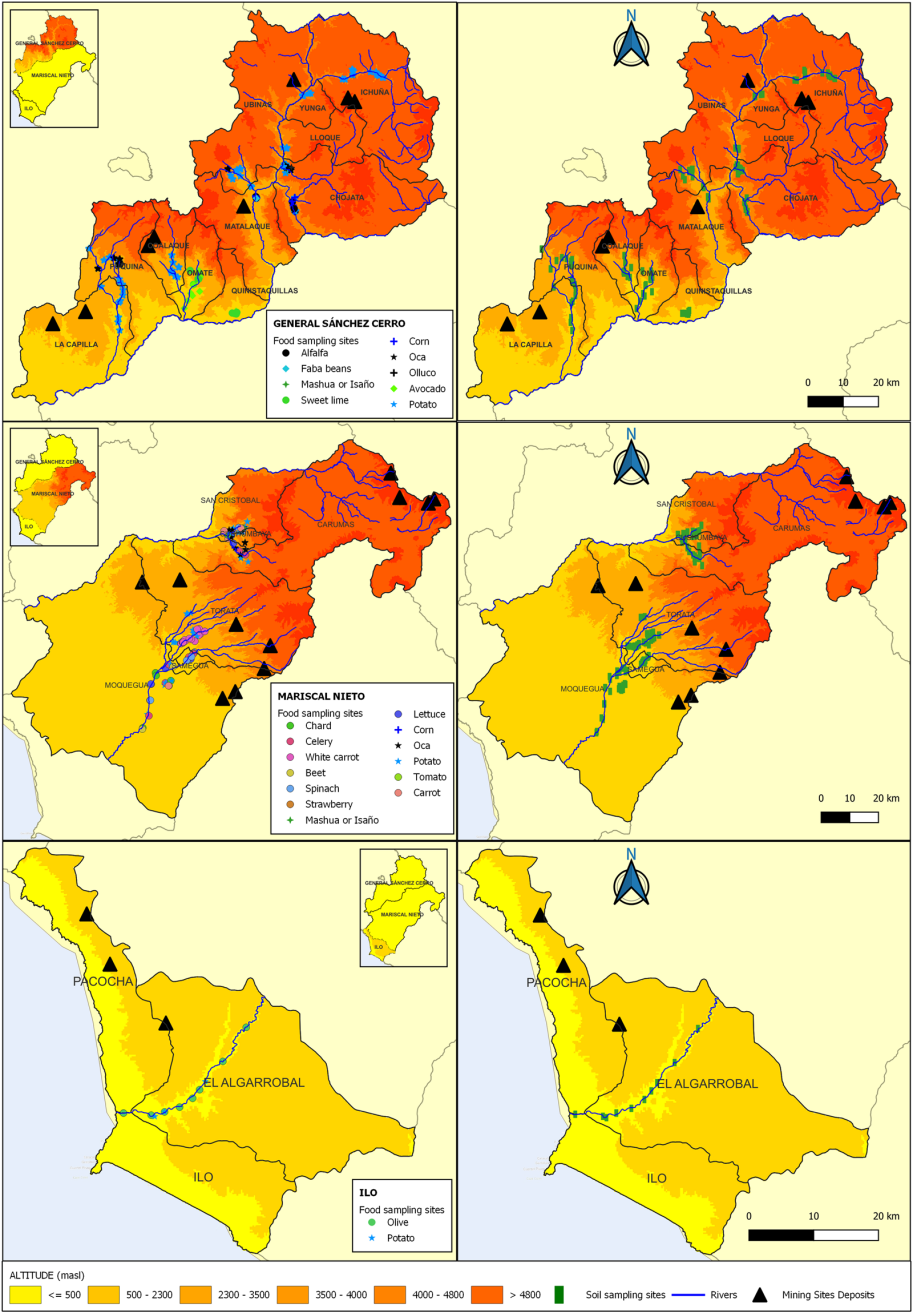
Spatial distribution of sampling points. The figure shows the location and altitudes of the farms where 341 food crop samples (left side) and soils (right side) were collected in the three provinces of the department of Moquegua: General Sánchez Cerro (1,539 to 3,934 m.a.s.l.), Mariscal Nieto (964 to 3,864 m.a.s.l.) and Ilo (9 to 357 m.a.s.l.). The food crops chosen are the most representative of each province and are important for the local diet.

Figure 3 shows the sampling sites in the three provinces of the department of Moquegua.

### Sample collection

Samples were collected in 2021 under the harvest season. Plants were selected at random from each field, to obtain the most representative sample possible of the land in question. UTM coordinates of the sampling fields were obtained using a handheld GPS (Garmin eTrex 10). The soils and the edible part of vegetables samples were collected from the same sampling points. Peru’s national guide for soil sampling was taken as a basis for this activity^54^. Depending on farm characteristics, the sampling points followed a diagonal and zigzag pattern, except for permanent plantations (avocado, olive and sweet lime), where they followed a S and W pattern. To obtain a significant sample, each one was a composite of at least 5 or 15 subsamples, for temporary and permanent crops, respectively. Plants from which the samples of interest were extracted had to have completed their vegetative period.

At each subsampling site, vegetable samples were removed with approximately 100 g of the soil in which the plant was growing. Soil samples were taken at random from the upper horizon (0–25 cm) and materials such as stone fragments, thick roots, organic residue and insects were removed. Both food crop and soil samples were mixed to form composite samples of about 1 kg each. The samples were placed in airtight polyethylene bags, which were labelled and transported to the laboratory. Exceptionally, three parcels provided samples of two food crops grown in the same soil (sample pairs 49–50, 204–205 and 259–260). Furthermore, at some sampling sites the food crops were not fully mature, so only soil samples were taken.

This sampling method has been used in other similar studies to quantify the concentration of heavy metals in tubers and roots^25^, leafy vegetable^55,56^, tomato^56^, and fruit trees^57^.

### Laboratory analyses

All analyses were performed at the ALS LS analytical laboratory, in Lima, Peru. The portion of food crop analysed varied, and is shown in the dataset. In some cases, the complete sample of the edible portion was used when this was the usual way of consuming them in that area. This was the case with tubers, leaf vegetables, carrots, beet, strawberries and tomatoes. In other cases, only one part was chosen; such as celery stems, avocado and olive pulp, and the peel of sweet lime because it has been shown that accumulation of As, Cd and Pb is higher in peel than in pulp^57^.

One part of the food crops, mainly tubers and corn, 31 HMM were analysed by inductively coupled plasma mass spectrometry (ICP-MS), using the EPA 200.3/EPA 6010B method validated in 2016 for vegetable tissue^58,59^. Another part of the samples were used to quantify As, Cd and Pb only, by atomic absorption spectrometry (AAS), as recommended by FAO in the General Methods of Analysis for Contaminants in foods^60^. For this we used the Mexican Official Standard NOM-117-SSA1-1994^61^. Both ICP-MS and AAS are two commonly-used methodologies for determining HMM in food^62^. HMM in the soil samples were analysed by inductively coupled plasma optical emission spectrometry (ICP-OES), using the EPA 3050 B method, Rev. 2 December/EPA 6010 D Rev. 5. July. 2018^63,64^. Quality control protocols for the instruments and methods were carried out by ALS LS laboratory^65^. The dataset specifies the method used for analysing each sample.

Table 1 presents the list of elements analysed with their respective quantification limits (LOQ) for food crops and soils and detection limits (LOD) for soils.

**Table 1:** Elements analysed, quantification limits (LOQ) and detection limits (LOD) presented in the dataset.

FOOD CROPS		SOILS		
Element	LOQ (mg/kg)	Element	LOD (mg/kg)	LOQ (mg/kg)
Aluminum (Al)	0.2	Aluminum (Al)	3.0	10.0
Antimony (Sb)	0.006	Antimony (Sb)	4.0	10.0
Arsenic (As)	0.01 (0.1*)	Arsenic (As)	3.6	5.5
Barium (Ba)	0.01	Barium (Ba)	0.3	1.0
Beryllium (Be)	0.0034	Beryllium (Be)	1.0	2.0
Bismuth (Bi)	1.0	Cadmium (Cd)	0.3	0.5
Boron (B)	25.0	Calcium (Ca)	1.5	2.5
Cadmium (Cd)	0.0012 (0.01*)	Chromium (Cr)	1.0	2.0
Calcium (Ca)	4.4	Cobalt (Co)	1.0	2.0
Chromium (Cr)	0.008	Copper (Cu)	0.8	2.5
Cobalt (Co)	0.0024	Iron (Fe)	2.5	6.0
Copper (Cu)	0.016	Lead (Pb)	3.0	5.0
Iron (Fe)	0.24	Magnesium (Mg)	3.0	17.0
Lead (Pb)	0.006 (0.038*)	Manganese (Mn)	2.0	10.0
Lithium (Li)	2.5	Molybdenum (Mo)	0.6	3.0
Magnesium (Mg)	5.2	Nickel (Ni)	1.0	2.0
Manganese (Mn)	0.022	Potassium (K)	3.5	10.0
Molybdenum (Mb)	0.004	Selenium (Se)	2.2	10.0
Nickel (Ni)	0.006	Silver (Ag)	0.9	1.7
Phosphorus (P)	150	Sodium (Na)	12.0	20.0
Potassium (K)	22.0	Thallium (Tl)	4.0	9.0
Selenium (Se)	0.04	Vanadium (V)	0.7	2.0
Silver (Ag)	0.0016	Zinc (Zn)	0.6	2.0
Sodium (Na)	3.6			
Strontium (Sr)	0.042			
Thallium (Tl)	0.0014			
Tin (Sn)	0.008			
Titanium (Ti)	0.018			
Uranium (U)	0.0008			
Vanadium(V)	0.004			
Zinc (Zn)	0.08			

*The value in brackets is the LOQ (quantification limit) of the AAS method. In all the other cases, the values expressed as LOQ or LOD (detection limit) are those for the ICP-MS (for food crop analysis) and ICP-OES (for soil analysis) methods.

## Data Records

All data records described in this article are publicly and freely available for download from Figshare^66^ repository. Our dataset provides results for concentrations of 31 HMM in 19 vegetable foods and 23 HMM in agricultural soils collected in an altitude range of 9 to 3,934 m.a.s.l. The list of HMM is given in Table 1. The dataset includes a total of 13,828 observations and provides information for each sample on the geographic location of the 341 sampling sites (province, district, farm sector, date taken), coordinates (altitude, latitude, longitude), portion of food crop analysed and analysis method.

## Technical Validation

In order to give official standing to our results, the chemical analyses were performed at the ALS LS analytical laboratory in Lima, Peru. This laboratory is accredited by the Instituto Nacional de Calidad del Peru - INACAL (National Quality Institute of Peru) in line with Peruvian Technical Standard NTP-ISO/IEC 17025, which establishes the general requirements for the competence of testing and calibration. This standard was drawn up by the International Standards Organisation (ISO) for evaluating conformity, and is approved by the national branches of the ISO and the International Electrotechnical Commission (IEC).

All the sample identification information in the dataset is duly recorded on a sampling card, including the personal data of the farm owners. Each sample in the dataset has a an assessment report issued by the laboratory and digital copies are available upon request.

The dataset is particularly valuable as it provides the initial reference values for HMM concentrations in food crops and agricultural soils within a traditional mining region, specifically focusing on copper exploration. As this dataset can be used as a baseline for future studies, it is worth pointing out some considerations that must be taken into account, both to plan the sample collection stage during HMM monitoring and to interpret the results based on a comparison with Peruvian regulations.

First, as is characteristic of the Andean countries in general, the geography of the department of Moquegua is quite complex and challenging for taking samples in the field; due to the difficulty of getting to farms from the roads, gradients, altitude variation and climate. This is probably the reason for the lack of records of HMM in food crops and agricultural soils, thus demonstrating the importance and novelty of this research into the dynamics of HMM in relation to altitude. Especially at higher altitudes (above 3,000 m.a.s.l.) food crops, mainly tubers and corn, are harvested only in March to April, which is the rainy season. Therefore sample collection depends on the weather and seasonality of agricultural production.

Secondly, in Peru there is no national regulation governing the maximum permissible limits of HMM in food, but the National Agrarian Health Service (SENASA) considers the Codex reference values and European Union regulations to be valid. Agricultural soils have Environmental Quality Standards (ECA), where reference values are established for arsenic (50 mg/kg), barium (750 mg/kg), cadmium (1.4 mg/kg), and lead (70 mg/kg)^67^. However it is important to take into account that prevention values are not given and that it would be interesting to analyse other countries’ standards, since these are an important decision-making tool when implementing soil protection policies.

## Data Availability

No custom code was generated for this work.
